# Implementation of the Flipped Classroom Combined with Problem-Based Learning in a Medical Nursing Course: A Quasi-Experimental Design

**DOI:** 10.3390/healthcare10122572

**Published:** 2022-12-19

**Authors:** Meixuan Chi, Naijuan Wang, Qing Wu, Ming Cheng, Chenya Zhu, Xiaohua Wang, Yunying Hou

**Affiliations:** 1 Nursing Department, The First Affiliated Hospital of Soochow University, Suzhou 215006, China; 2 School of Nursing, Suzhou Medical College of Soochow University, Suzhou 215123, China

**Keywords:** flipped classroom, problem-based learning, small private online course, medical nursing

## Abstract

Background: Medical Nursing is one of the most important core courses in nursing education, and the emergence of the flipped classroom has made up for the shortcomings of traditional teaching and improved the effectiveness of teaching. However, it is worth exploring how to maximize the effect of students’ self-study before class while making full use of classroom teaching to promote the cultivation of students’ abilities, so that the flipped classroom can have a maximal teaching effect. Therefore, this study explored the effect of a flipped-classroom teaching mode based on a small private online course (SPOC) combined with problem-based learning (PBL) in a course of Medical Nursing. Methods: Nursing undergraduates from the years 2018 (control group) and 2019 (experimental group), respectively, used the traditional lecture method and the flipped-classroom teaching mode based on a SPOC combined with PBL. The teaching effect was evaluated by teaching-mode-recognition evaluation, critical thinking measurement, and academic achievement. Results: The scores of teaching-mode recognition evaluated by the students in the experimental group were higher than those in the control group in the following five aspects: “helping to improve learning interest” (*p* = 0.003), “helping to improve autonomous learning ability” (*p* = 0.002), “helping to improve communication and cooperation ability” (*p* < 0.001), “helping to cultivate clinical thinking” (*p* = 0.012), and “helping to promote self-perfection and sense of achievement” (*p* = 0.001). Compared with the control group, the score on the “analytical ability” dimension of the Critical Thinking Disposition Inventory in the experimental group was higher (*p* = 0.030). The excellent rates of the final theoretical examination (*p* = 0.046) and comprehensive case analysis (*p* = 0.046) in the experimental group were higher than those in the control group. Conclusions: The flipped-classroom teaching mode based on a SPOC combined with PBL can promote students’ abilities of autonomous learning, communication and cooperation, and clinical and critical thinking; improves their academic performance; and is recognized and welcomed by them. However, to extend the flipped-classroom teaching model of a SPOC combined with PBL to other nursing education courses, more optimization and evaluation are required.

## 1. Introduction

With the increasing demands for health services and the complexity of health care systems, nurses are expected to acquire appropriate abilities and skills to make reasonable decisions and handle real-life situations [1]. This requires the introduction of relevant teaching methods in nursing education.

Among the many educational methods used to foster the correlation between the educational and clinical performance of nursing students, problem-based learning (PBL) is one of the most widely employed [2]. Grounded in the principle that knowledge is constructed and shaped in social contexts and using problem cases as vehicles for learning [3], PBL has been proved to improve nursing students’ application of theory lessons in clinical practice, learning motivation, critical thinking, self-learning capabilities, and satisfaction with teaching [4,5,6]. Unfortunately, no single education strategy is perfect, and one of the disadvantages of PBL is that knowledge acquired through PBL is less organized than knowledge acquired through traditional learning [7].

As a result of recent developments in the fields of internet technologies, social networks, and learning management systems, the flipped classroom is being used more and more by educators these days. The flipped classroom is one of the blended learning methodologies that combine e-learning and face-to-face classroom technique, which is intended to improve the efficacy of classroom learning by allowing students to control the timing and pace of their online learning and maximize their opportunities for active learning by engaging in class discussions and collaborative exercises in the company of peers and instructors [8]. Previous studies have demonstrated that, compared to traditional lecture-based classrooms, flipped classrooms are more effective in improving academic performance and appraisal on the course as well as developing engagement and higher-level thinking skills (critical, logical, reflective, metacognitive, and creative) in nursing students [9,10,11]. 

In various disciplines, the flipped classroom has been used in different models. In nursing education, as one of the most important teaching methods with the advantage of improving nursing students’ clinical performance and higher-level thinking ability, PBL can be used as an in-class activity in the flipped classroom. On the other hand, to some extent, pre-class e-learning in the flipped classroom can compensate for the disadvantage of PBL in the systematization of knowledge acquisition. However, very few studies have investigated the combination of a flipped classroom and PBL in nursing education [10]. 

A Medical Nursing course is one of the most important core courses in nursing programs. The course aims to build students’ knowledge systems of caring for patients with medical diseases and cultivate students’ professional skills to assess, analyze, and solve clinical problems. Traditional in-class lectures are not conducive to the cultivation of students’ thinking abilities and are unable to promote the connection of theoretical knowledge and clinical practice skills. In view of the advantages and benefits of PBL and the flipped classroom, these methods may be suitable for changing the traditional classroom to meet the needs of the students, and a combination of these methods may produce better outcomes in the teaching of Medical Nursing. However, to our knowledge, there are no studies evaluating the effectiveness of the pedagogy that combines the flipped classroom with PBL in the teaching of Medical Nursing. 

Therefore, the purpose of this study is to (1) implement a flipped classroom combined with e-learning and PBL in a Medical Nursing course and (2) evaluate the students’ academic performance, critical thinking, and appraisal to verify the effectiveness of the combination. The study is significant in that the flipped classroom used in combination with PBL expands the existing models of the flipped classroom in nursing education and might provide a reference for nursing educators to improve students’ performance. 

## 2. Materials and Methods

### 2.1. Study Design 

This study adopted a quasi-experimental design with a control group.

### 2.2. Setting and Participants

Since there is only one teaching class for each year of the nursing major in the college, the control group consisted of 33 undergraduate students from the class of 2018, and the experimental group consisted of 57 undergraduate students from the class of 2019. Four students in the Class of 2019 had incomplete questionnaires, so the experimental group ended up including 53 students.

### 2.3. The Flipped-Classroom Teaching Model of a SPOC—Small Private Online Course (SPOC) Combined with PBL

**Figure 1:**
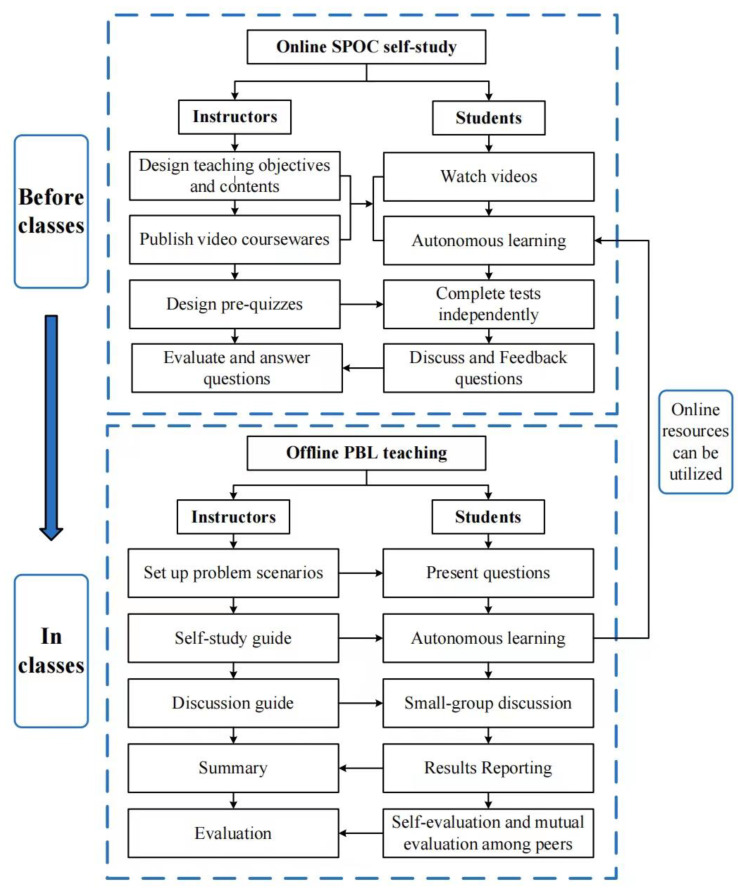
Schematic diagram of the online and offline flipped-classroom teaching model of SPOC combined with PBL.

Using the teaching platform resources, the online and offline flipped-classroom teaching model of Medical Nursing combining SPOC with PBL is mainly designed in two modules: Online SPOC pre-class teaching and offline PBL in-class teaching, as shown in Figure 1.

#### 2.3.1. Teaching Steps

The teachers create teaching videos, determine teaching materials, and prepare teaching tasks based on course knowledge points; arrange the students to complete learning tasks with the help of the SPOC online learning platform; and conduct PBL group in-class teaching in the PBL classroom so that the students can carry out learning activities such as discussion and sharing in the classroom.

#### 2.3.2. Online Pre-Class SPOC Teaching Session

Each student enters the SPOC of Medical Nursing for autonomous learning by logging in to the wisdom tree online course platform with his or her student ID and password. The online course resources are divided into two sections: mandatory learning content and optional learning content before the class. The mandatory learning content before the class refers to the core knowledge points that the teachers think the students must master before PBL learning, and the students need to watch videos, do homework, and take online tests independently before the class. The optional learning content is mainly the teaching videos that the students need to watch independently in the process of PBL learning. The students can participate in the platform’s interactive forum, online communication platform, and other teaching platforms for consultation if they encounter problems in the process of online learning.

#### 2.3.3. Offline In-Class PBL Teaching Session

(1) Building a PBL case base

Before the class begins, the teachers carefully select and adapt real clinical cases together with medical and nursing staff according to the requirements of the teaching objectives to form a PBL-teaching case library. Each case includes at least 2 acts.

(2) Formation of PBL student groups

Each group has six to ten pupils. Students with different characteristics are reasonably matched based on gender, learning capacity, personality, and other factors.

(3) Conducting PBL teaching

The students work in small groups, each with an instructor, and are provided with the case in acts and sections. The students present the “problems” to be solved around the case scenario and discuss them in small groups to propose ways to solve the “problems”. For those “problems” that cannot be solved with their current knowledge base, the group members determine a plan of action and engage in autonomous learning to retrieve information. During the self-study process, the students can continue to log in to the SPOC learning platform of Medical Nursing to study and consult relevant materials and can also use other information retrieval channels. After one week, the group members reconvene to communicate and share the information that they have obtained, re-examine the “problem” based on the new knowledge they have learned, propose solutions to the problem, and so on, until all the “problems” presented in the case have been solved.

(4) Student report and teacher summary

The students report the results of the case discussion in groups in various forms, such as PPT, diagrams, role plays, etc. The teacher remarks on and summarizes the reports from each group, briefly summarizes the knowledge in correlation with the problems encountered by the students in their autonomous learning, and explains and answers questions about the students’ error-prone and confusing areas, as well as common difficult problems.

(5) Evaluation

Through self-evaluation and mutual evaluation among the students, the students evaluate their abilities in autonomous learning, problem analysis, communication, teamwork, etc. The teachers also evaluate the students’ aforementioned abilities and provide timely feedback to the students.

### 2.4. Implementation of the Flipped-Classroom Teaching Model of a SPOC Combined with PBL

Except for the different teaching methods, the experimental and control groups had the same teaching contents and hours, and both were taught by the same teaching team. With regard to (1) the control group, the teaching method of theoretical lectures combined with ward-based bedside internship was adopted. The theoretical lectures were based on the traditional teaching method of classroom lectures, with the textbook as the center, and the classroom lectures were conducted in accordance with the sequence of the textbook and the requirements of the syllabus by using multimedia and courseware. With regard to (2) the experimental group, the flipped-classroom teaching model of an online SPOC combined with offline in-class PBL teaching was adopted for some chapters, see above. All PBL instructors received PBL tutor training from Fudan University Shanghai Medical College and obtained qualification certificates. The probation hours, contents, forms, and teachers of the experimental group were the same as those of the control group, and there was no difference in the teaching contents and forms of other courses between the two groups.

### 2.5. Data Collection

A self-evaluation questionnaire on critical thinking skills was given to the two groups of students before the course began, and a self-administered teaching-model-approval questionnaire and a self-evaluation questionnaire on critical thinking skills were given to the two groups of students at the end of the course. The students were instructed with uniform instructions to fill out the questionnaires, and the questionnaires were collected on the spot. The teaching-model-recognition evaluation questionnaire [12,13,14,15] was developed by the research team based on the literature from other studies and two rounds of Delphi expert consultation. The questionnaire had nine items: helping to master the key contents, helping to master knowledge systematically, helping to improve learning interest, improving learning efficiency, helping to improve the ability to obtain information, helping to improve autonomous learning ability, helping to enhance communication and cooperation ability, helping to cultivate clinical thinking, and helping to promote self-perfection and sense of achievement. The questionnaire was scored on a 5-point Likert scale, ranging from “completely disagree” (1 point) to “completely agree” (5 points), with higher scores indicating greater acceptance of the teaching model. The Critical Thinking Disposition Inventory-Chinese Version (CTDI-CV) was developed by Professor Meici Peng, School of Nursing, The Hong Kong Polytechnic University [16]. The CTDI-CV contains 70 items measuring 7 dimensions: truth-seeking, open-mindedness, analytical ability, systematization, self-confidence in critical thinking, intellectual curiosity, and cognitive maturity. The items of the questionnaire were scored on a 6-point Likert scale, ranging from “strongly disagree” (1 point) to “strongly agree” (6 points), with 30 items on a positive scale and 40 items on a negative scale. Total scores range from 70 to 420, with higher scores indicating better critical-thinking ability. Cronbach’s α coefficient was 0.90.

The assessment of academic achievements consists of three parts: a theoretical examination, a comprehensive case analysis, and a clinical probation report, each with a score of 100. Among them, the theoretical examination questions include multiple-choice questions, short-answer questions, and case-analysis questions of a single disease. A comprehensive case analysis is a complex case analysis including at least 2 diseases. The grade of the probation report is the average score of each disease probation report, including medical history summary, physical examination, psychosocial assessment, laboratory test items and positive results, nursing diagnosis, and nursing measures. The theoretical examinations and case analyses were reviewed by the teaching assistants, and the probation reports were reviewed by the teaching staff of the corresponding clinical departments, without knowledge of the students’ grouping. Before the review, the course lecturer unifies the training review requirements and rules.

### 2.6. Data Analysis

The data were analyzed using SPSS, version 19.0. Categorical variables were expressed as frequency (n) and percentage (%), and numerical data were expressed as mean (M) and standard deviation (SD). An independent-samples *t*-test and a chi-square test were used to verify that there were no differences between experimental and control groups in terms of age and gender. For the teaching-mode-recognition evaluation and critical-thinking ability, we compared them between the two groups by using independent-sample *t*-tests. Chi-square tests were used to compare the excellent rates of achievement assessments between the two groups. *p*-values of less than 0.05 were considered significant.

## 3. Results

### 3.1. Characteristics of Participants

A total of 86 students were included in the study. Table 1 shows the demographic characteristics of the participants. The mean age was 20.3 (0.684) for the control group and 20.57 (0.694) for the experimental group. There were 8 (24.20%) males and 25 (75.80%) females in the control group. The experimental group had 11 (20.80%) males and 42 (79.20%) females. The experimental group did not statistically differ from the control group in terms of age and gender (*p* > 0.05).

### 3.2. The SPOC’s Online Learning Results 

All students in the experimental group completed the task of watching the required videos, and the average viewing rate of the selected videos was 85.9%. The correct rate of each online test question was 86~97%. A total of 13 (34%) of the students answered all test questions correctly, and 32 (60%) of the students correctly answered more than 80% of the questions.

### 3.3. The Teaching-Mode-Recognition Evaluation

A total of 52 (98%) of the students thought that the flipped-classroom teaching model of a SPOC combined with PBL helped to master the key contents, systematically master knowledge, improve autonomous learning ability, enhance communication and cooperation ability, and promote self-improvement. A total of 51 (96%) of the students thought it helped to improve learning interest, improve the ability to obtain information, and develop clinical thinking. In total, 50 (94%) of the students thought it could improve learning efficiency. The scores of teaching-mode recognition evaluated by the students in the experimental group were higher than those in the control group in the five aspects of “helping to improve learning interest” (*p* = 0.003), “helping to improve autonomous learning ability” (*p* = 0.002), “helping to improve communication and cooperation ability” (*p* < 0.001), “helping to cultivate clinical thinking” (*p* = 0.012), and “helping to promote self-perfection and sense of achievement” (*p* = 0.001). The results are shown in Table 2.

### 3.4. Critical-Thinking Ability

Between the two groups, there were no statistically significant differences in each dimension or in the total scores of critical-thinking ability at the beginning of the Medical Nursing course (*p* > 0.05). After the course, the score on the “analytical ability” dimension in the experimental group was higher than that of the control group (*p* = 0.030), but there were no differences in the rest of the dimensions or in the total scores (*p* > 0.05). The results are shown in Table 3.

### 3.5. Achievement Assessment

The excellent rates of the final theoretical examination (*p* = 0.047) and the comprehensive case analysis (*p* = 0.046) in the experimental group were higher than those in the control group. There was no difference in the rate of excellence in the clinical probation report (*p* = 0.056). The results are shown in Table 4.

## 4. Discussion

The PBL teaching method can develop students’ skills in various areas, such as problem-solving, time management, teamwork, higher-level thinking, and the ability to obtain and evaluate information and use it to construct knowledge flexibly [17,18]. However, a drawback of the PBL teaching method is the lack of systematically acquired information [19,20], and the lack of effective teaching resources can greatly affect the effect of PBL teaching. In this study, the SPOC online course platform was used to fully meet the students’ needs for self-study before and after classes. It was combined with offline PBL teaching to fully integrate the optimal online and offline teaching resources for the flipped-classroom model, which improved the students’ problem-solving and clinical-thinking ability while promoting the systematization and integrity of knowledge acquisition and achieved good teaching results.

### 4.1. The Flipped-Classroom Teaching Model of a SPOC Combined with PBL Was Recognized by the Students

The traditional teaching model based on a teacher’s lecture can give full play to the teacher’s organizational role in classroom teaching. However, it ignores students’ initiative, creativity, and cognitive subject role, which is not conducive to improving students’ interest in learning and autonomous learning ability. Furthermore, there is insufficient teacher-student and student-student communication, and the classroom atmosphere is not active in the traditional teaching mode [21]. The flipped classroom based on a SPOC and PBL not only promotes the students’ autonomous learning of basic concepts and knowledge before the classes by using online resources, but especially in the process of finding answers and solving problems based on PBL cases, students also need to independently retrieve and learn relevant knowledge. This is conducive to promoting students’ subjective initiative and improving their autonomous learning ability, in line with the results of Zeng [22] and Zhao [23]. As an important core educational course of the nursing profession, Medical Nursing not only emphasizes the mastery of basic knowledge and concepts but also pays more attention to the cultivation of students’ clinical-thinking and practical problem-solving abilities. Based on clinical real situation cases, PBL learning encourages students to find, analyze, and solve problems in the process of independent exploration, which is conducive to promoting the cultivation of students’ clinical-thinking ability and shortening the distance between school and clinic [24]. We found that students can obtain a great sense of self-satisfaction in the process of independent analysis and problem solving, which is beneficial for promoting students’ self-improvement and increasing their interest in learning. Moreover, the university stage is a key period of life development, and having good communication and cooperation skills is the basis for nursing students to improve their comprehensive quality, clinical nurse-patient communication skills, and health-care-team cooperation skills [25]. In our study, group learning and interactive discussion in the PBL learning process were conducive to the cultivation of the students’ communication and cooperation skills.

### 4.2. The Flipped-Classroom Teaching Model of a SPOC Combined with PBL Could Improve Students’ Critical-Thinking Ability to a Certain Extent

Critical thinking in nursing is a purposeful and meaningful process of self-regulated judgment and reflective reasoning about nursing phenomena and problems, which enables nurses to make rational nursing decisions [26]. Critical-thinking ability is one of the basic qualities and primary core abilities required for clinical nursing practice and nursing research, and cultivating critical-thinking ability in undergraduate nursing students is regarded as the most important function of nursing education and is a basic requirement for professional nursing education [27]. In this study, through the combination of a SPOC and offline PBL teaching, the students were guided to use high-quality and rich teaching resources to conduct problem inquiry, analysis, and discussion based on real and incomplete clinical situation cases. The students finally solved the problems implied in the cases. Simultaneously, the students were guided to judge the scientificity of the information obtained by other group members and consciously reflected on the process of problem-solving. All of these were conducive to the development of critical-thinking skills. As Zhang et al.’s study demonstrated, PBL teaching supported by wireless networks and mobile intelligent terminals resulted in higher scores in the students’ total critical-thinking ability and the four dimensions of truth-seeking, analytical ability, self-confidence in critical thinking, and cognitive maturity than the traditional classroom lecture method [28]. However, this study found that the students scored higher than the control group only on the analytical ability dimension, which may be related to the small number of students, the insufficient sample size, and the insufficient number of credit hours in the new teaching model. It is necessary to increase the number of teaching classes, credit hours, and the teaching contents covered in the flipped classroom of a SPOC combined with PBL in future teaching to further observe its effect on the development of critical-thinking ability.

### 4.3. The Flipped-Classroom Teaching Model of a SPOC Combined with PBL Helped to Improve the Students’ Academic Achievement

In the flipped-classroom teaching model of a SPOC combined with PBL, the growth of the students’ learning initiative and academic achievement could be aided by the stimulation of learning interest, self-improvement, sense of accomplishment, and the increase in autonomous learning ability. This study was limited by the number of teaching classes and students, so it was not possible to conduct a parallel control trial. However, this study took the form of comparing one teaching class in each of the two grades before and after. Because the difficulty of the test paper can not be guaranteed to be completely consistent, the excellent rate of the examination score was used as an index to evaluate the academic achievement between experimental and control groups. The results of the study showed that the experimental group had significantly higher excellent rates in both the theoretical examination and the comprehensive case analysis than the control group. Previous studies have also shown that the teaching model of a SPOC combined with flipped classrooms could improve students’ performance in the theoretical assessment of Medical Nursing [29]. However, distinct from previous studies, this study focused on offline teaching using the PBL teaching method in addition to flipped-classroom teaching using SPOC online resources, which not only enabled the students to master theoretical knowledge systematically, but also improved the students’ abilities to analyze and solve problems as well as their clinical-thinking ability. The excellent rate of the students’ comprehensive case analysis scores was significantly higher than that of the control group.5. Limitations 

This study had some limitations. First, there was a limited sample size. Second, there was only one teaching class per grade, so it was not possible to conduct a concurrent controlled trial. Third, after the end of the course, we did not follow up on the effectiveness of the course evaluation for a long period of time.

## 5. Conclusions 

We developed a flipped-classroom teaching model of a SPOC combined with PBL in a Medical Nursing course and showed that this teaching model can promote students’ abilities of autonomous learning, communication and cooperation, and clinical and critical thinking; can improve their academic performance; and is recognized and welcomed by them. We suggest that nursing educators adopt teaching strategies that promote autonomous learning, make full use of online learning resources, and conduct dual teaching online and offline to actively respond to the slogan of higher education reform and promote the development of nursing education. However, to extend the flipped-classroom teaching model of a SPOC combined with PBL to other nursing education courses, more optimization and evaluation are required.

## Figures and Tables

**Table 1:** Characteristics of participants.

Characteristics	Control Group (n = 33)		Experimental Group (n = 53)		*p* Value
	Mean	SD	Mean	SD	
Age	20.3	0.684	20.57	0.694	0.089 ^a^
Gender	N	%	N	%	0.791 ^b^
Male	8	24.20%	11	20.80%	
Female	25	75.80%	42	79.20%	

^a^ The independent-sample *t*-test. ^b^ The chi-square test.

**Table 2:** Comparison of students’ recognition evaluation of the teaching model between two groups.

Items	Control Group (n = 33)	Experimental Group (n = 53)	t	*p* Value ^a^
Helping to master the key contents	4.39 (0.70)	4.23 (0.76)	1.028	0.741
Helping to master knowledge systematically	4.24 (0.83)	4.30 (0.82)	0.309	0.815
Helping to improve learning interest	3.58 (1.17)	4.37 (0.84)	3.724	0.003
Improving learning efficiency	4.03 (0.85)	4.18 (1.02)	0.691	0.393
Helping to improve the ability to obtain information	3.97 (1.02)	4.39 (0.84)	2.097	0.167
Helping to improve autonomous learning ability	3.67 (1.24)	4.44 (0.80)	3.583	0.002
Helping to enhance communication and cooperation ability	3.58 (1.28)	4.46 (0.73)	4.164	<0.001
Helping to cultivate clinical thinking	3.55 (1.25)	4.25 (0.89)	3.085	0.012
Helping to promote self-perfection and sense of achievement	3.73 (1.26)	4.42 (0.73)	3.317	0.001

^a^ The independent-sample *t*-test.

**Table 3:** Comparison of students’ critical-thinking ability between two groups at the end of the course.

Items	Control Group (n = 33)	Experimental Group (n = 53)	t	*p* Value ^a^
Truth-seeking	34.45 (6.41)	34.89 (8.33)	0.262	0.513
Open-mindedness	39.94 (7.60)	39.77 (6.70)	0.109	0.818
Analytical ability	30.79 (6.34)	36.49 (9.20)	3.146	0.030
Systematization	38.33 (6.15)	38.77 (7.04)	0.298	0.163
Self-confidence in Critical thinking	41.73 (5.99)	40.74 (6.50)	0.717	0.633
Intellectual curiosity	45.94 (6.59)	45.16 (6.82)	0.530	0.655
Cognitive maturity	42.79 (4.94)	45.21 (4.84)	2.270	0.542
Total score	273.97 (21.74)	281.04 (26.66)	1.293	0.245

^a^ The independent-sample *t*-test.

**Table 4:** Comparison of students’ achievement assessments between two groups.

Items	Control Group (n = 33)		Experimental Group (n = 53)		χ^2^	*p* Value ^b^
	N	%	N	%		
theoretical examination results ≥ 85 points	5	15.15%	18	33.96%	3.949	0.047
comprehensive case analysis results ≥ 85 points	6	18.18%	20	37.74%	3.989	0.046
clinical probation report results ≥ 85 points	19	57.58%	40	75.47%	3.642	0.056

^b^ The chi-square.

## Data Availability

The datasets generated during this study are available by mail to corresponding authors upon reasonable request.
